# Cyclic Thermomechanical Elasto-Viscoplasticity Implementation Using User Material Interface

**DOI:** 10.3390/ma18112512

**Published:** 2025-05-27

**Authors:** Marko Nagode, Simon Oman, Jernej Klemenc, Domen Šeruga

**Affiliations:** University of Ljubljana, Faculty of Mechanical Engineering, Aškerčeva 6, SI-1000 Ljubljana, Slovenia

**Keywords:** finite element method, thermomechanical loading, elasto-viscoplasticity, fatigue, creep, dissipated energy

## Abstract

The paper introduces a user material for Abaqus, detailing the modeling of elasto-viscoplasticity under diverse thermomechanical conditions. Converting constitutive equations into a robust code requires extensive efforts to solve numerous crucial numerical challenges. In addition to deriving the equations, detailing the code is also crucial for an efficient implementation of a rheological model. The algorithm for multiaxial Prandtl operator approach presented here provides both. The subroutines of the numerical code are explained in detail and solutions to ensure numerical stability are demonstrated. The multiaxial Prandtl operator approach allows a simple and effective calculation of fatigue damage, creep damage, e.g., or dissipated energy using available uniaxial methods. To demonstrate practical application, the paper illustrates the usefulness of the code by analyzing perforated plates under tension–compression and shear loading. This contribution enriches the computational modeling of elasto-viscoplasticity for the finite element method.

## 1. Introduction

The appearance of computers has laid the path for enabling a comprehensive understanding of the behaviour of engineering structures [1,2,3,4,5,6,7,8,9,10]. Macro parameters, such as Young’s modulus, Poisson’s ratio, and uniaxial compressive or tensile strength can now be simulated with numerical models [1,2,3]. Similarly, failure modes, effects of particle size distribution, or the relationship between the load and axial displacement and the design of complex cross-sectional properties (e.g., circular or square concrete-filled stub columns) can be examined [4,5]. A variety of important engineering tasks, such as structural performance assessment of historical masonry buildings during their service life, fatigue behaviour of complex welded structures, design of variable stiffness composites, or even virtual testing for investigation of the shear behaviour of cross-ply composites can now be performed due to powerful computer-based analyses [6,7,9,11]. New theories are also emerging due to the availability of computer simulations that only existed in a theoretical form a few decades ago [10,12]. Although analytical solutions offer a decision-making tool for suitability of mechanical design and can incorporate rather complex constitutive modelling, only simpler shapes can usually be considered for manipulation with the resulting mathematical expressions [13,14,15]. Lyu et al. [13] set analytical solutions for axial stress, strain, and displacement on the base of kinematic equilibrium and both elastic and thermo-elastic constitutive relationships considering the pile–soil interface and proved applicability of the behaviour prediction of energy piles under various loads. Šeruga et al. [14] showed that the analytical expression of a simple rigidly supported beam, subjected to a vertical displacement at one of the supports, already provides a substantial level of complexity regardless of the variable cross-section and other varying geometric properties. Similarly, Peng et al. [15] provided an analytical three-dimensional model for a coupled magneto-mechanical behaviour of a rectangular-shaped actuator made of ferromagnetic shape memory alloy. Initially used only for a few discretisations, the finite element method (FEM) is currently used to handle hundreds of thousands of finite elements and solve millions of equations in a matter of hours [16,17,18,19].

The finite element method has the distinguishing feature that an arbitrary shape of a continuum can be described by a set of discrete (finite) elements, which makes it excellently applicable to complex changes of geometric forms of modern complex mechanical components [19]. The approximation of the, e.g., unknown stress–strain distribution function in the case of a structural analysis is built within each finite element where the vertices of the finite elements (nodes) represent the degree of freedom [20]. Guo and Zhang used FEM recently to create a finite element model of a whole human body with a detailed lumbar spine [19]. Similarly, Mizushima and Kinoshita [21] used a detailed finite element model to simulate the behaviour of a several-story reinforced concrete building. A high-resolution FE model was also used by Chen et al. [16] to reproduce a barge–bridge collision. However, for solution of certain problems, e.g., to solve elliptic interface problems with non-homogeneous jump conditions on surfaces or to characterise the stress singularity at the crack tip of a notch, other numerical methods, e.g., the finite difference method or the finite block method can outperform the finite element method [22,23]. Interface problems in particular can be successfully addressed by evolved or even conglomerate methods, joining several numerical routines, such as the extended finite element method [24], Nitsche extended finite element method [25,26], weak Galerkin finite element method [27,28], or CutFEM [29].

Although analyses considering elastic material behaviour are sufficient for components that operate in the fatigue endurance area, modern design features allow for elastoplasticity occurrence around geometric details, which requires special attention when it comes to accurate stress–strain response modelling or lifetime prediction [30,31,32,33,34]. Recently, Chen and Izzuddin [31] presented a simplified finite strain plasticity model for metallic applications that significantly reduces the computational demand for two numerical problems—an impact of a cylindrical bar onto a rigid frictionless plate at a high speed and a cylindrical rod subjected to uniaxial extension. Furthermore, Jrad et al. [35] investigated ceramic/metal functionally graded material shells with nonlinear elastoplastic material properties and implemented the algorithm into ABAQUS/Standard via a user material subroutine (UMAT). Pirzadeh et al. [33] suggested an elastoplastic peridynamic formulation with the capability of the elastoplastic law to describe either isotropic, kinematic, or mixed hardening of the material. Similarly, Oliveira and Penna [32] presented a formulation that generalises frequently used elastoplastic models and tested it on several benchmark cases, e.g., bending of a cantilever beam or a V-notched bar and stretching of a perforated plate. However, variable temperature during operation can make the operating conditions more demanding, so consideration of both the changing mechanical and thermal operating conditions requires dedicated material models that can handle the phenomena occurring in the material during the loading. Some of the latest publications regarding variable thermomechanical loads include a study of Ahn et al. [36] regarding a new multiphysics mode synthesis for the thermomechanical vibration problem. Furthermore, Dörr et al. [37] implemented a coupled thermomechanical approach for finite element forming simulation of continuously fiber-reinforced semi-crystalline thermoplastics into FE solver Abaqus. Moreover, a micromechanical procedure based on a thermo-visco-hyperelastic constitutive model has been proposed and developed through the finite element method by Moradi et al. [38] for evaluating the thermomechanical behaviour of ternary acrylate-based shape memory polymer nanocomposites containing carbon nanotube and graphene nanoplatelet hybrids.

The typical material models suitable for FE implementation and simulation of elastoplastic or elasto-viscoplastic material response during thermomechanical loading follow a well-accepted elastic predictor–plastic corrector iteration routine [39,40,41,42,43,44]. Chen et al. [39] recently presented a damage coupled elastoplastic constitutive model of marine high-strength steels under low cycle fatigue loadings. Moreover, Eghtesad et al. [40] implemented an elasto-visco-plastic fast Fourier transform crystal plasticity model in the implicit FE method of Abaqus standard through a UMAT subroutine. Park et al. [41] coupled a crystal plasticity FE method to simulate heterogeneous deformation and a probabilistic cellular automata approach with a dynamic recrystallization model to simulate microstructure evolution using the predictor–corrector routine. Viscoplastic models typically follow the Chaboche framework [45], which originates from the Armstrong–Frederick time-independent elastoplastic material model [46] considering a nonlinear kinematic hardening law and a power law to describe the time-dependent material properties [47]. Another typical material model frequently used in finite element solvers is the Besseling model, which enables simulations of stress–strain behaviour considering multilinear kinematic hardening [48]. On the contrary, the constitutive equations for the multiaxial Prandtl operator approach were recently suggested by Nagode et al. [49,50] as a closed-form solution, independent from the iterative procedure to determine the exact temperature-dependent elastoplastic or elasto-viscoplastic solution during given variable thermomechanical loading. Advantageous computational power under complex loading paths, description of the cyclic curve by either uniaxial Ramberg–Osgood or Armstrong–Frederick relations [51] and Norton or a more complex law to describe the viscoplastic behaviour [52], multilinear kinematic hardening during cyclic loading [53], a small number of material parameters gained from basic uniaxial tests, and their simple determination [30,54] are the main attributes of the developed approach.

Specifically, the gain of computational power in the case of the closed-form Prandtl operator approach [49,50] is the main motivation for the present work. The novelty as compared to the previous studies is to offer a complete source code for the ABAQUS/Standard implementation of the multiaxial elasto-viscoplastic Prandtl operator approach via a user material subroutine (UMAT) taking into account a Ramberg–Osgood description of the elastoplastic material law, Norton viscoplastic law, and multilinear kinematic hardening during cyclic loading. The UMAT code is thoroughly explained and discussed to provide an understanding of the calculation algorithm. The subroutines of the numerical code are explained in detail, and solutions to ensure numerical stability are demonstrated. Finally, the material model is tested on typical examples for multiaxial stress–strain simulations considering several variable thermomechanical load histories.

## 2. Theoretical Fundamentals

In this section, we present a brief overview of a newly developed temperature-dependent elasto-viscoplastic non-unified constitutive model. The model assumes knowledge of the stress tensor $\sigma_{ij}^{\left(1\right)}$, the strain tensor $\varepsilon_{ij}^{\left(1\right)}$, the temperature $T^{\left(1\right)}$, and the time $t^{\left(1\right)}$ for the previously accepted equilibrium state denoted as (1). It takes into account the strain tensor increment $∆\varepsilon_{ij}$, the temperature increment ∆T, and the time increment ∆t. Additionally, small deformations are assumed. Only mechanical strains are considered in the calculation, and the stress calculation depends on the temperature-dependent stress–strain relation. However, thermal strains can be taken into account as additional load, and their consideration is well-documented in Abaqus documentation.

### 2.1. Initialisation

The procedure starts with the initialisation of the variables. It is assumed that there is no initial residual strain, resulting in $n_{q}\geq 1$ radial elastoplastic back-strains $E_{\rho l}^{\left(1\right)}$ in the deviatoric plane being

(1) $$E_{\rho l}^{\left(1\right)}=0,\;l=1,\ldots ,n_{q}$$

The stress tensor $\sigma_{ij}^{\left(1\right)}$, the radial stress in the deviatoric plane $s_{\rho }^{\left(1\right)}$, the radial elastoplastic strain in the deviatoric plane $e_{\mathrm{ep},\rho }^{\left(1\right)}$, the maximum radial elastoplastic strain in the deviatoric plane $e_{\mathrm{ep},\max}^{\left(1\right)}$, the viscoplastic strain tensor $\varepsilon_{\mathrm{vp},ij}^{\left(1\right)}$, the effective elastoplastic strain $\varepsilon_{\mathrm{ep},\mathrm{eff}}^{\left(1\right)}$, and the effective viscoplastic strain $\varepsilon_{\mathrm{vp},\mathrm{eff}}^{\left(1\right)}$ are also initially set to zero.

### 2.2. Viscoplasticity Assessment

Given the known stress tensor and radial stress in the deviatoric plane for state (1), we can determine the deviatoric stress tensor as

(2) $$s_{ij}^{\left(1\right)}=\sigma_{ij}^{\left(1\right)}-\frac{1}{3}\sigma_{kk}^{\left(1\right)}\delta_{ij}$$

and the effective stress as

(3) $$\sigma_{\mathrm{eff}}^{\left(1\right)}=\sqrt{\frac{3}{2}}s_{\rho }^{\left(1\right)}$$

Next, we determine the effective viscoplastic strain rate using, for example, the law of perfect viscoplasticity with elastic domain [55]:

(4) $${\dot{p}}^{\left(1\right)}=\mathrm{sign}\left(\sigma_{\mathrm{eff}}^{\left(1\right)}\right){\langle \frac{|\sigma_{\mathrm{eff}}^{\left(1\right)}|-k\left(T^{\left(1\right)}\right)}{K(T^{\left(1\right)})}\rangle }^{N(T^{\left(1\right)})}$$

Here, the temperature-dependent material parameters $K(T^{\left(1\right)})$, $N(T^{\left(1\right)})$, and the elastic limit $k(T^{\left(1\right)})$ are assumed to be known. The angular brackets represent a conditional operator 〈x〉=x if x>0, and 〈x〉=0 if x≤0. The viscoplastic strain tensor increment is then determined as [56]

(5) $$∆\varepsilon_{\mathrm{vp},ij}=\frac{3}{2}\frac{s_{ij}^{\left(1\right)}}{|\sigma_{\mathrm{eff}}^{\left(1\right)}|}∆\varepsilon_{\mathrm{vp},\mathrm{eff}}$$

where

(6) $$∆\varepsilon_{\mathrm{vp},\mathrm{eff}}={\dot{p}}^{\left(1\right)}∆t$$

represents the effective viscoplastic strain increment, calculated using Euler’s explicit forward integration scheme. The viscoplastic strain tensor and the effective viscoplastic strain in the current state (2) are then given by

(7) $$\varepsilon_{\mathrm{vp},ij}^{\left(2\right)}=\varepsilon_{\mathrm{vp},ij}^{\left(1\right)}+∆\varepsilon_{\mathrm{vp},ij}$$

and by

(8) $$\varepsilon_{\mathrm{vp},\mathrm{eff}}^{\left(2\right)}=\varepsilon_{\mathrm{vp},\mathrm{eff}}^{\left(1\right)}+∆\varepsilon_{\mathrm{vp},\mathrm{eff}}$$

respectively. The corresponding radial viscoplastic strain in the deviatoric plane for the current state is then

(9) $$e_{\mathrm{vp},\rho }^{\left(2\right)}=\sqrt{\frac{3}{2}}\varepsilon_{\mathrm{vp},\mathrm{eff}}^{\left(2\right)}$$

It is worth noting that the law of perfect viscoplasticity in Equation (4) can be replaced by alternative viscoplasticity laws, such as the hyperbolic sine [57] or the exponential form [58] or others.

### 2.3. Elastoplasticity Assessment

The elastoplastic strain tensor increment is determined as follows:

(10) $$∆\varepsilon_{\mathrm{ep},ij}=∆\varepsilon_{ij}-∆\varepsilon_{\mathrm{vp},ij}$$

Next, the deviatoric elastoplastic strain tensor increment is obtained by subtracting one-third of its trace:

(11) $$∆e_{\mathrm{ep},ij}=∆\varepsilon_{\mathrm{ep},ij}-\frac{1}{3}∆\varepsilon_{\mathrm{ep},kk}\delta_{ij}$$

The second and third deviatoric elastoplastic strain-invariant increments are

(12) $$∆{\tilde{J}}_{2}=\frac{1}{2}∆e_{\mathrm{ep},ij}∆e_{\mathrm{ep},ji}$$

and

(13) $$∆{\tilde{J}}_{3}=\frac{1}{3}∆e_{\mathrm{ep},ij}∆e_{\mathrm{ep},jk}∆e_{\mathrm{ep},ki}$$

The radial elastoplastic strain increment, as expressed in [59], is given by

(14) $$∆\rho_{e}=\sqrt{2∆{\tilde{J}}_{2}}$$

If $∆{\tilde{J}}_{3}>0$, the radial elastoplastic strain increment is positive. Conversely, if $∆{\tilde{J}}_{3}<0$, it is negative. However, for pure shear when $∆{\tilde{J}}_{3}=0$, the model cannot determine the sign of $∆\rho_{e}$ solely based on $∆{\tilde{J}}_{3}$. The radial elastoplastic strain in the deviatoric plane for the current state is now calculated as

(15) $$e_{\mathrm{ep},\rho }^{\left(2\right)}=e_{\mathrm{ep},\rho }^{\left(1\right)}+∆\rho_{e}$$

This yields the effective elastoplastic strain increment:

(16) $$∆\varepsilon_{\mathrm{ep},\mathrm{eff}}=\sqrt{\frac{2}{3}}∆\rho_{e}$$

and the effective elastoplastic strain in the current state (2):

(17) $$\varepsilon_{\mathrm{ep},\mathrm{eff}}^{\left(2\right)}=\varepsilon_{\mathrm{ep},\mathrm{eff}}^{\left(1\right)}+∆\varepsilon_{\mathrm{ep},\mathrm{eff}}$$

Thus, $∆\varepsilon_{\mathrm{ep},\mathrm{eff}}$ represents the effective elastoplastic strain increment, and $\varepsilon_{\mathrm{ep},\mathrm{eff}}^{\left(2\right)}$ represents the effective elastoplastic strain in the current state. To enhance computational efficiency, the maximum radial elastoplastic strain in the deviatoric plane is also calculated using the following expression:

(18) $$e_{\mathrm{ep},\max}^{\left(2\right)}=\max(e_{\mathrm{ep},\max}^{\left(1\right)},|e_{\mathrm{ep},\rho }^{\left(2\right)}\left|\right)$$

Next, the radial elastoplastic back-strains $E_{\rho l}^{\left(2\right)}$ are computed for the current state using the following equation:

(19) $$E_{\rho l}^{\left(2\right)}=\max\left\{e_{\mathrm{ep},\rho }^{\left(2\right)}-q_{l},\min\left\{e_{\mathrm{ep},\rho }^{\left(2\right)}+q_{l},E_{\rho l}^{\left(T\right)}\right\}\right\},\;l=1,\ldots ,n_{q}\wedge e_{\mathrm{ep},\max}^{\left(2\right)}\leq q_{l}$$

where

(20) $$E_{\rho l}^{\left(T\right)}=\left\{\begin{array}{ll} 0, & \;\mathrm{if}\;\alpha_{l}\left(T^{\left(1\right)}\right)\alpha_{l}\left(T^{\left(2\right)}\right)<0\\ \frac{\alpha_{l}\left(T^{\left(1\right)}\right)}{\alpha_{l}\left(T^{\left(2\right)}\right)}E_{\rho l}^{\left(1\right)}, & \;\mathrm{if}\;\left|\alpha_{l}\left(T^{\left(1\right)}\right)\right|<\left|\alpha_{l}\left(T^{\left(2\right)}\right)\right|\\ E_{\rho l}^{\left(1\right)}, & \;\mathrm{otherwise}\; \end{array}\right.$$

The material parameters $q_{l}$ and $\alpha_{l}$, which represent the yield radii and the temperature-dependent Prandtl densities, respectively, are explained in detail in [49].

The radial stress in the deviatoric plane $s_{\rho }^{\left(2\right)}$ for the current state can then be calculated as follows:

(21) $$s_{\rho }^{\left(2\right)}=\sum_{l=1}^{n_{q}}s_{\rho l}^{\left(2\right)}=\underset{e_{\mathrm{ep},\max}^{\left(2\right)}\leq q_{l}}{\sum_{l=1}^{n_{q}}}\alpha_{l}\left(T^{\left(2\right)}\right)E_{\rho l}^{\left(2\right)}$$

which represents the Prandtl operator [60]. Consequently, the corresponding radial stress increment in the deviatoric plane is given by

(22) $$∆\rho_{s}=s_{\rho }^{\left(2\right)}-s_{\rho }^{\left(1\right)}$$

Furthermore, the effective stress increment is

(23) $$∆\sigma_{\mathrm{eff}}=\sqrt{\frac{3}{2}}∆\rho_{s}$$

and the effective stress for the current state (2) is

(24) $$\sigma_{\mathrm{eff}}^{\left(2\right)}=\sigma_{\mathrm{eff}}^{\left(1\right)}+∆\sigma_{\mathrm{eff}}$$

### 2.4. Stress Tensor Assessment

The exact closed-form expression for the stress tensor increment is composed as follows:

(25) $$∆\sigma_{ij}=2\mu^{*}\left(T^{\left(2\right)}\right)∆\varepsilon_{\mathrm{ep},ij}+\lambda^{*}\left(T^{\left(2\right)}\right)∆\varepsilon_{\mathrm{ep},kk}\delta_{ij}$$

where the Lamé constants are given by

(26) $$2\mu^{*}\left(T^{\left(2\right)}\right)=\frac{∆\rho_{s}}{∆\rho_{e}}=\frac{2}{3}\frac{∆\sigma_{\mathrm{eff}}}{∆\varepsilon_{\mathrm{ep},\mathrm{eff}}}$$

and by

(27) $$\lambda^{*}\left(T^{\left(2\right)}\right)=K\left(T^{\left(2\right)}\right)-\frac{2}{3}\mu^{*}\left(T^{\left(2\right)}\right)$$

Here, $K(T^{\left(2\right)})$ represents the bulk modulus at temperature $T^{\left(2\right)}$. Finally, the stress tensor in the current state (2) is obtained by adding the stress tensor increment to the previous stress tensor:

(28) $$\sigma_{ij}^{\left(2\right)}=\sigma_{ij}^{\left(1\right)}+∆\sigma_{ij}$$

### 2.5. Consistent Material Jacobian

The consistent material Jacobian for the elasto-viscoplastic material model for $0<|∆\rho_{s}/∆\rho_{e}|<\infty$ can be written as

(29) $$\begin{array}{cc} \delta ∆\sigma_{ij} & =2\mu^{*}\left(T^{\left(2\right)}\right)\delta ∆\varepsilon_{\mathrm{ep},ij}+\lambda^{*}\left(T^{\left(2\right)}\right)\delta_{ij}\delta ∆\varepsilon_{\mathrm{ep},kk}\\ & +\frac{3}{2}\left(\alpha^{*}\left(T^{\left(2\right)}\right)-2\mu^{*}\left(T^{\left(2\right)}\right)\right)\eta_{ij}\eta_{kl}\delta ∆\varepsilon_{\mathrm{ep},kl} \end{array}$$

where $\eta_{ij}$ is attained by dividing Equation (11) by Equation (16):

(30) $$\eta_{ij}=\frac{2}{3}\frac{∆e_{\mathrm{ep},ij}}{∆\varepsilon_{\mathrm{ep},\mathrm{eff}}}$$

and $\alpha^{*}\left(T^{\left(2\right)}\right)$ is

(31) $$\alpha^{*}\left(T^{\left(2\right)}\right)=\sum_{\begin{array}{c} l=1\\ e_{ep,max}^{\left(2\right)}\leq q_{l} \end{array}}^{n_{q}}\left\{\begin{array}{ll} \alpha_{l}\left(T^{\left(2\right)}\right), & \;\mathrm{if}\;e_{ep,\rho }^{\left(2\right)}>E_{\rho l}^{\left(T\right)}+q_{l}\;\mathrm{or}\;e_{ep,\rho }^{\left(2\right)}<E_{\rho l}^{\left(T\right)}-q_{l}\\ 0, & \;\mathrm{otherwise}\; \end{array}\right.$$

For $|∆\rho_{s}/∆\rho_{e}|\to 0$ or $|∆\rho_{s}/∆\rho_{e}|\to \infty$, Equation (29) can be simplified as

(32) $$\delta ∆\sigma_{ij}=2\mu^{*}\left(T^{\left(2\right)}\right)\delta ∆\varepsilon_{\mathrm{ep},ij}+\lambda^{*}\left(T^{\left(2\right)}\right)\delta_{ij}\delta ∆\varepsilon_{\mathrm{ep},kk}$$

For a detailed derivation of the equations of Section 2, see [49].

## 3. Implementation

The multiaxial Prandtl operator approach, which takes elastoplastic and elasto-viscoplastic material behaviour into account, was implemented in the finite element solver Abaqus, which allows users to implement user material via the UMAT subroutine in the Fortran language. Since the implementation itself was very arduous and many numerical problems had to be solved, this section aims to present the complete code and also to point out the possible places in the code that require further attention. The subroutines are listed and explained in detail in the order in which they appear in the code. However, it is assumed that the reader is familiar with the basics of Fortran and can also find information in the Abaqus help that is not directly related to the implementation of the material model.

### 3.1. Subroutine UEXTERNALDB

**Figure 1:**
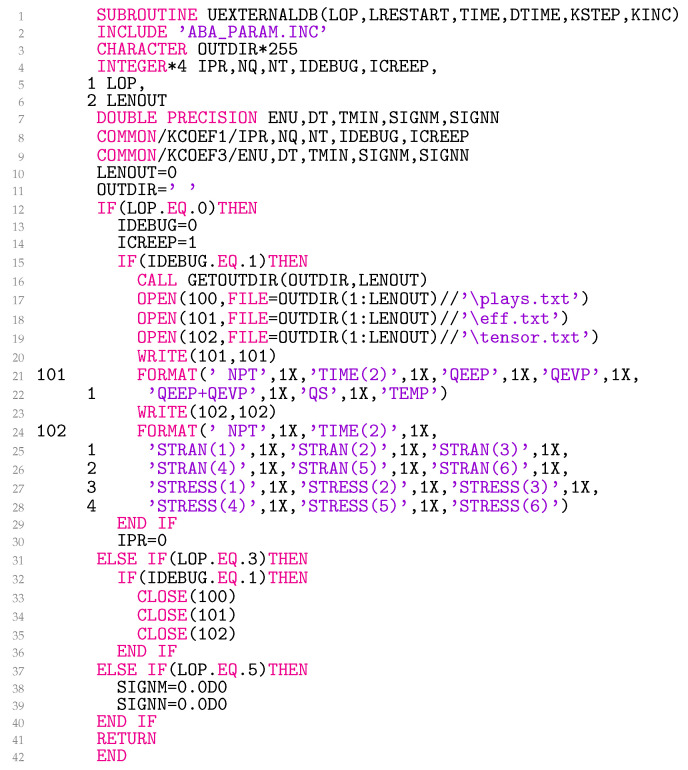
Coding for setting model-specific flags, opening and closing files.

The code in Figure 1 can be executed in two modes: IDEBUG=0 or IDEBUG=1. The first option is the fastest, as only one *.odb output is generated, which can be managed later in Abaqus CAE. In the second case, the model-specific information is output in three files: ‘plays.txt’, ‘eff.txt’ and ‘tensor.txt’. The IDEBUG=1 mode should be used with great caution as it can generate large files, but it can be helpful in validating and updating the model. It is usually advisable to use this option only for certain integration point number NPT and element number NOEL.

The files also contain information on the total time at the beginning of the current increment TIME(2), the radial elastoplastic strain in the deviatoric plane $e_{\mathrm{ep},\rho }^{\left(1\right)}$ (QEEP), the radial viscoplastic strain in the deviatoric plane $e_{\mathrm{vp},\rho }^{\left(1\right)}$ (QEVP) and the total radial strain in the deviatoric plane $e_{\rho }^{\left(1\right)}=e_{\mathrm{ep},\rho }^{\left(1\right)}+e_{\mathrm{vp},\rho }^{\left(1\right)}$ (QEEP+QEVP), the radial stress in the deviatoric plane $s_{\rho }^{\left(1\right)}$ (QS), and the temperature $T^{\left(1\right)}$ (TEMP) as well as the stress $\sigma_{ij}^{\left(1\right)}$ (STRESS) and strain $\varepsilon_{ij}^{\left(1\right)}$ (STRAN) tensors. The tensors are always returned in the order 11, 22, 33, 12, 13, 23.

The multiaxial Prandtl operator approach can be made elastoplastic when ICREEP=0 or elasto-viscoplastic for ICREEP=1. In the subroutine, IPR is set to 0 at the beginning of the analysis for LOP=0. In this way, the input data from the Abaqus input file *.inp are read only once in the subroutine UMAT explained in Section 3.5. At the beginning of each step, the common block variables SIGNM and SIGNN are set to zero. The variables are used to interrelate the integration points of all elements with respect to the sign of $∆{\tilde{J}}_{3}$, as explained in detail in Section 3.3.

### 3.2. Subroutine SDVINI

**Figure 2:**
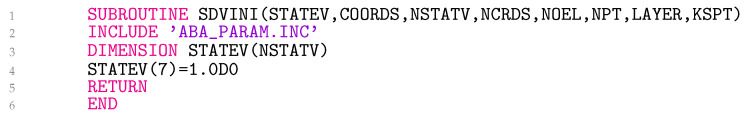
Coding of solution-dependent state variables.

The subroutine SDVINI, as depicted in Figure 2, assigns the value of 1.0 to STATEV(7) in order to ascertain the direction of the radial elastoplastic strain increment during the first step within the UMAT subroutine.

### 3.3. Subroutine KSIGN

**Figure 3:**
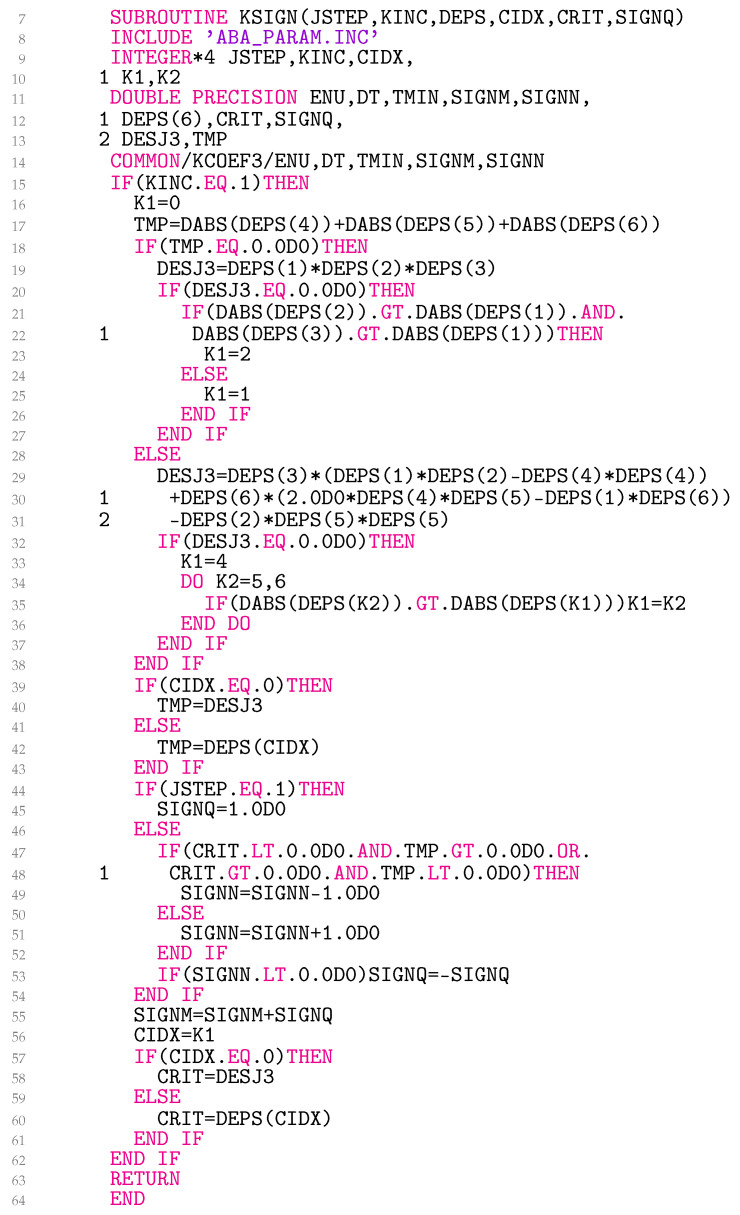
Coding the sign of the third deviatoric elastoplastic strain-invariant increment.

Although the calculation of the third deviatoric elastoplastic strain-invariant increment as outlined in Equation (13) may seem numerically straightforward, it became clear that the sign of $∆{\tilde{J}}_{3}$ poses significant numerical challenges. The complexity arises from the obvious need for a connection between the sign values at different integration points and elements. An endeavor to address the issue is illustrated in Figure 3.

Line 15 shows that the sign is fixed only at the beginning of the step, assuming that SIGNQ remains constant throughout the step. Lines 16–38 calculate $∆{\tilde{J}}_{3}$, which is represented in the code as DESJ3, where DEPS stands for the deviatoric elastoplastic strain tensor increment. Two cases are considered. If $∆e_{\mathrm{ep},ij}$ is diagonal, DESJ3 is calculated in line 19. If $∆{\tilde{J}}_{3}=0$, the integration point is in pure shear. In order to still be able to define the sign, we have linked the sign to the DEPS(K1) as listed in lines 20–27. For nondiagonal $∆e_{\mathrm{ep},ij}$, DESJ3 is calculated in lines 29–31. In the case of pure shear, the sign in lines 32–37 is associated with the absolute largest nondiagonal DEPS(K1). It can be further noted that K1 for pure shear is either 1, 2, 4, 5, or 6 and 0 otherwise. The value is stored in line 56 in the state variable CIDX.

In lines 39–43, TMP, which represents the current step, is linked to either DESJ3 or DEPS(CIDX). The state variable CRIT, on the other hand, retains the identical information from the previous step. By scrutinizing the signs of TMP and CRIT in lines 47 and 48, the common block variable SIGNN is computed either in line 49 or 51. Should SIGNN prove to be less than zero, the sign alteration occurs, as depicted in line 53. The variables SIGNM and SIGNN traverse all integration points and elements. In line 58 or 60, the state variable CRIT is then updated to assume the value of either DESJ3 or DEPS(K1).

It would indeed be better to determine the sign outside the UMAT subroutine. At present, however, this does not seem to be a feasible option within Abaqus, at least not in a straightforward way. The current code execution can therefore be influenced by the number of CPUs used.

### 3.4. Subroutine KPLAYS

**Figure 4:**
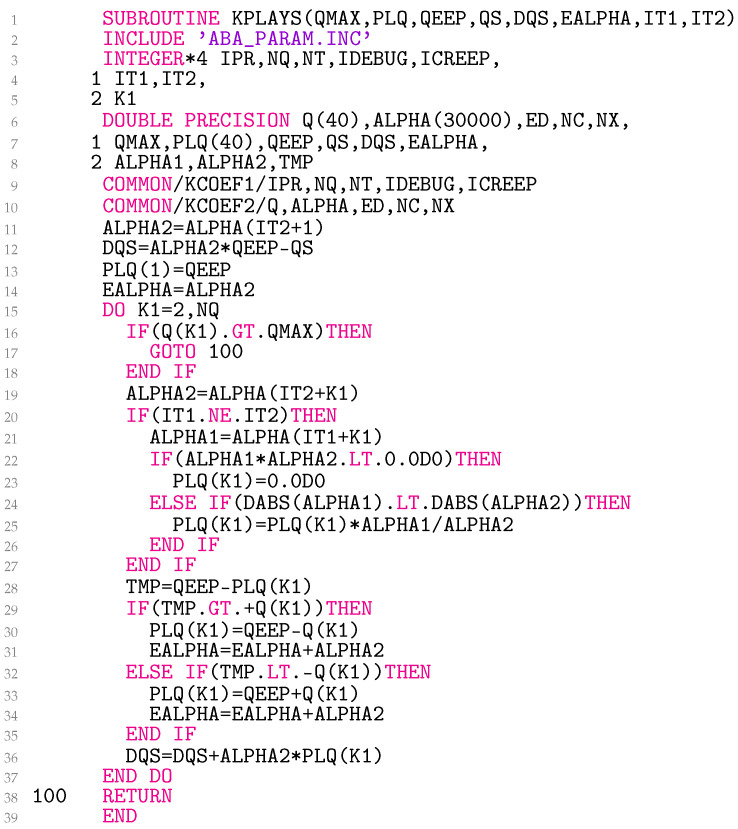
Coding of variables of the Prandtl operator.

The subroutine KPLAYS (Figure 4) takes as input the maximum radial elastoplastic strain $e_{\mathrm{ep},\max}^{\left(2\right)}$ (QMAX), the radial elastoplastic back-strains $E_{\rho l}^{\left(1\right)}$ (PLQ), $e_{\mathrm{ep},\rho }^{\left(2\right)}$ as QEEP, $s_{\rho }^{\left(1\right)}$ as QS, and the temperature index for the previous IT1 and the current IT2 state. The number of yield radii $n_{q}$ (NQ), the yield radii $q_{l}$ (Q) for $l=1,\ldots ,n_{q}$, and the temperature-dependent Prandtl densities $\alpha_{l}$ (ALPHA) are available in the subroutine as global variables. The material parameter Q is a static array with a maximum length of $n_{q}\leq 40$. The same applies to PLQ. ALPHA also represents a static array. Its length is currently limited to $n_{q}\times n_{T}\leq 30000$. The number of distinct temperatures $n_{T}$ (NT) is also a global variable, currently limited to 750. The array lengths can be adjusted, uniformly, in all subroutines. ALPHA1 and ALPHA2 contain $\alpha_{l}\left(T^{\left(1\right)}\right)$ and $\alpha_{l}\left(T^{\left(2\right)}\right)$, respectively.

The subroutine returns the radial elastoplastic back-strains $E_{\rho l}^{\left(2\right)}$ in PLQ, the radial stress increment in the deviatoric plane $∆\rho_{s}$ (DQS), and $\alpha^{*}\left(T^{\left(2\right)}\right)$ as EALPHA.

### 3.5. Subroutine UMAT

**Figure 5:**
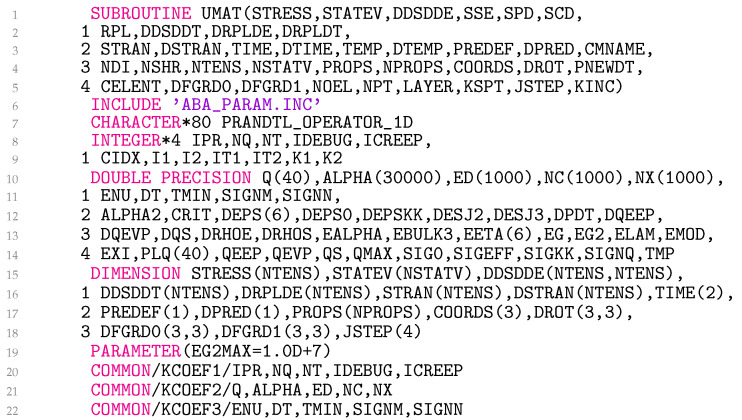
Declaration of variables of the user material model.

The subroutine UMAT is divided into six parts because of its length. The first five lines in Figure 5 list the input and output arguments in parentheses, as required by Abaqus. Any Abaqus/Standard user subroutine written in Fortran must include ’ABA_PARAM.INC’ as the first statement after the argument list. Lines 7–14 declare character, various integer, and real variables. Lines 15–18 declare arrays with their respective dimensions. Line 19 defines a single constant with the statement PARAMETER, which assigns specific values to the variables. Lines 20–22 declare the common blocks KCOEF1, KCOEF2, and KCOEF3, which allow variables to be shared by different parts of the programme.

**Figure 6:**
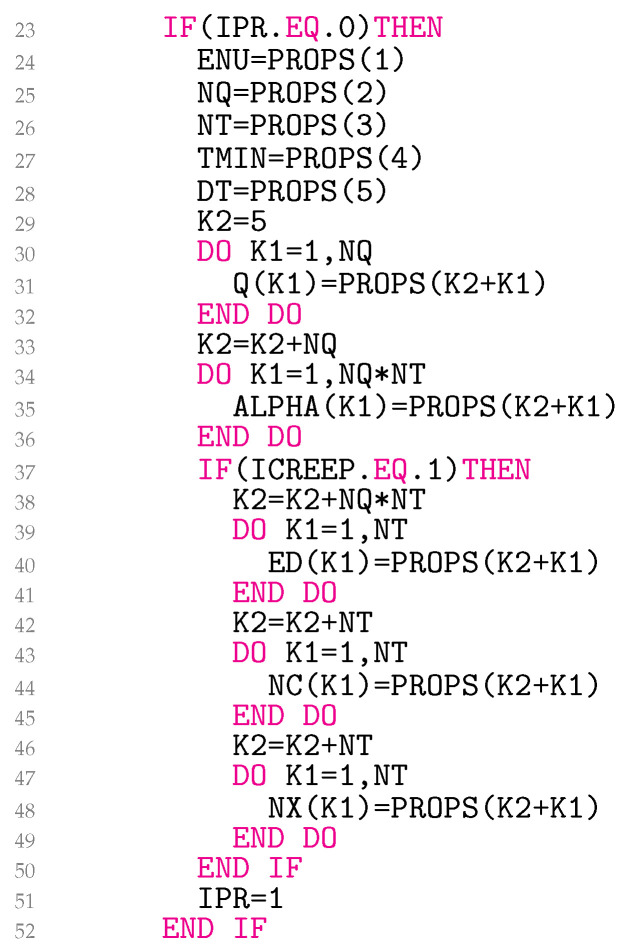
Reading out variables of the user material model.

The reading of the material parameters stored in the file *.inp is explained in Figure 6. The Poisson’s ratio ν in PROPS(1) is stored in the global variable ENU. The next values in the user-defined array of material constants PROPS associated with the user material are NQ and NT. The minimum test temperature $T_{\min}$ (TMIN) for which the material parameters are available and the corresponding temperature increment ∆T (DT) are read in lines 27–28. In lines 29–32, the yield radii $q_{l}$ (Q) for $l=1,\ldots ,n_{q}$ are read in Q. The first yield radius $q_{1}$ is always 0. Temperature-dependent Prandtl densities $\alpha_{l}\left(T_{k}\right)$, where $T_{k}=T_{\min}+∆T\left(k-1\right)$ for $l=1,\ldots ,n_{q}$ and $k=1,\ldots ,n_{T}$, are read in a one-dimensional array ALPHA in lines 33–36. This is done so that $n_{q}$ values for temperature $T_{1}=T_{\min}$ appear first in ALPHA, then $n_{q}$ values for $T_{2}$, and so on. The variable ends with $n_{q}$ values for $T_{n_{T}}=T_{\max}$.

For the law of perfect viscoplasticity with elastic domain, the elastic limit $k(T_{k})$ is read into ED for $k=1,\ldots ,n_{T}$. Similarly, $N\left(T_{k}\right)\log K(T_{k})$ is stored in NC and $N(T_{k})$ in NX. For other viscoplasticity laws, lines 38–49 may need to be changed. This part of the code is only called once because of the flag IPR.

**Figure 7:**
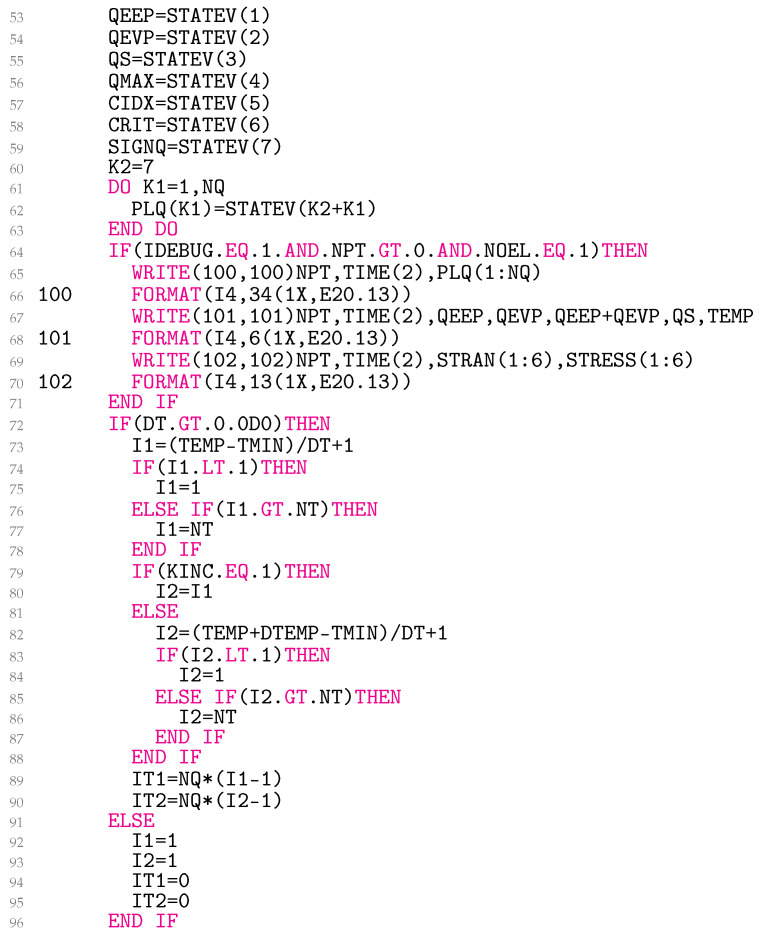
Reading solution-dependent state variables, writing to files and coding temperature indices.

Lines 53–63 in Figure 7 are needed to read the solution-dependent state variables in QEEP, QEVP, QS, QMAX, CIDX, CRIT, the sign of $∆\rho_{e}$ (SIGNQ), and PLQ. In lines 64–71, the data about the previously accepted equilibrium state (1) are written in ‘plays.txt’, ‘eff.txt’, and ‘tensor.txt’ when DEBUG=1. In this particular case, the data for element number 1 are stored at all integration points. Line 64 may be changed as required.

The block of lines 73–90 is only executed if the temperature increment DT in the file *.inp is greater than zero. The variables I1 and I2 contain the indices of the previously accepted temperature and the current temperature. The indices enable fast access of proper values from one-dimensional arrays ED, NC, and NX. The indices IT1 and IT2, on the other hand, provide quick access to the corresponding values from the array ALPHA. For isothermal conditions, set DT in *.inp to zero. In this instance, the indices take the values from lines 92–95. In the first increment, line 80 is executed. This simplification affects the code’s ability to converge and has minimal impact on the results.

**Figure 8:**
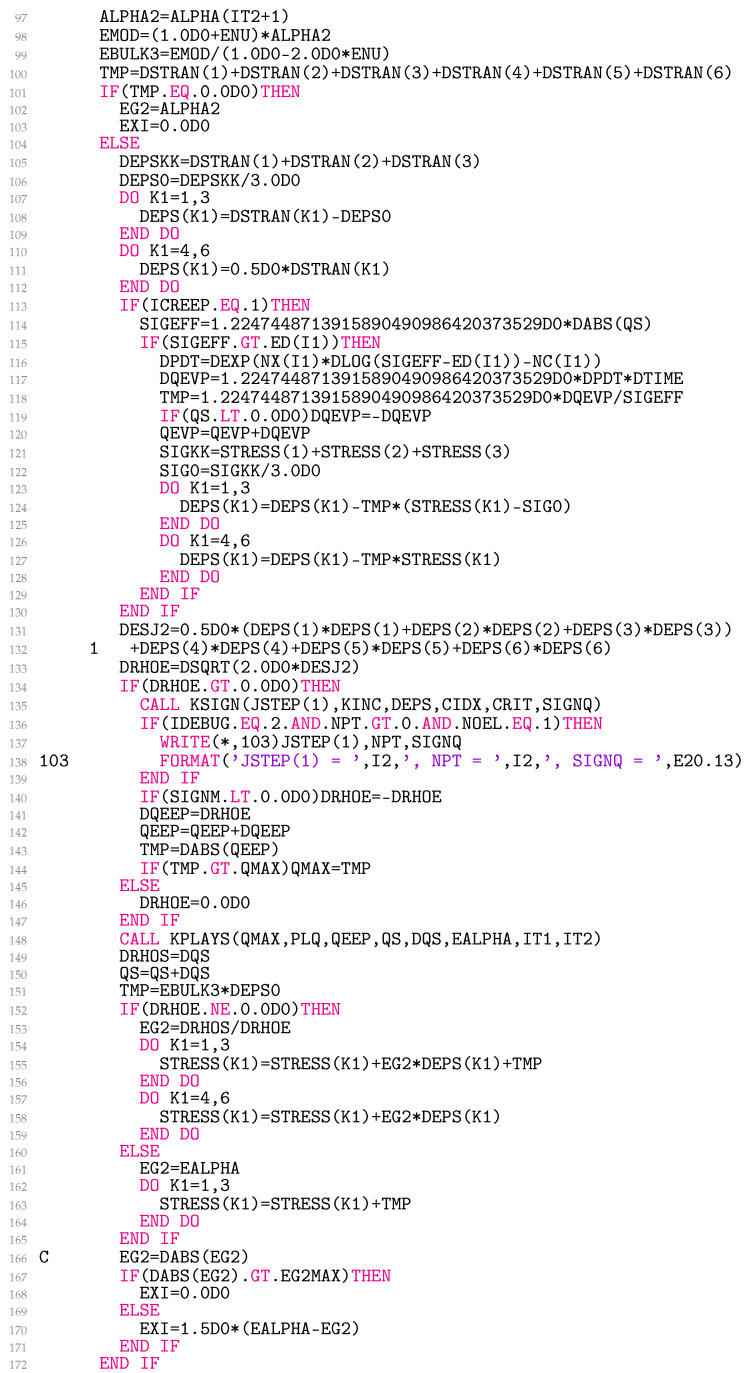
Coding of the first and the second UMAT call.

The ALPHA2 in line 97 of Figure 8 is twice the shear modulus $2G(T^{\left(2\right)})$, EMOD stands for the Young’s modulus $E(T^{\left(2\right)})$, and EBULK3 is three times the bulk modulus $3K(T^{\left(2\right)})$. The UMAT is called twice on the first iteration of each increment, once for assembly and once for recovery, also for linear analysis. Thereafter, it is called once per iteration, combining assembly and recovery. During the first call, the initial stiffness matrix of the model is formulated. In this call, the increment of the strain tensor, $∆\varepsilon_{ij}$, is set to 0, which is detected in line 100. The line could be made safer by summing up the absolute strain values instead of merely calculating the sum of the tensor components. However, we prioritized speed over safety. It is also important to emphasise that the constitutive model presented does not require the two-call scheme.

Lines 102–103 involve the preparation of variables required for the calculation of the elastic stiffness matrix, which takes place in lines 173–199. The variables EG and EG2 stand for $G(T^{\left(2\right)})$ and $2G(T^{\left(2\right)})$, respectively, and ELAM is $\lambda^{*}\left(T^{\left(2\right)}\right)$ for $\mu^{*}\left(T^{\left(2\right)}\right)=G\left(T^{\left(2\right)}\right)$. The variable EXI controls whether Equation (29) or Equation (32) is used for the consistent material Jacobian calculation.

In lines 105–112, the increment of the deviatoric strain tensor is calculated. The UMAT works with engineering shear strains; therefore, the DSTRAN in line 111 is multiplied by 0.5. In line 114, the absolute value of the effective stress $\sigma_{\mathrm{eff}}^{\left(1\right)}$ is calculated and stored in the variable SIGEFF. In lines 117 and 120, the radial viscoplastic strain increment $∆e_{\mathrm{vp}}$ and the radial viscoplastic strain $e_{\mathrm{vp},\rho }^{\left(2\right)}$ are determined. In lines 121–128, the deviatoric elastoplastic strain tensor increment $∆e_{\mathrm{ep},ij}$ is assessed.

In lines 131–133, the increment of the second deviatoric elastoplastic strain invariant, $∆{\tilde{J}}_{2}$, and the radial elastoplastic strain increment, $∆\rho_{e}$, are resolved.

The subroutine KSIGN is crucial for determining the sign of $∆\rho_{e}$. If the loading is monotonically increasing or decreasing, lines 135 and 140 can be safely omitted. When the loading consists of several steps in which the external force, displacement, or temperature change direction, lines 135 and 140 are requested.

Lines 136–139 can write important values related to the sign of $∆\rho_{e}$ to the file *.log. Line 142 calculates the radial elastoplastic strain in the deviatoric plane $e_{\mathrm{ep},\rho }^{\left(2\right)}$. Line 144 updates $e_{\mathrm{ep},\max}^{\left(2\right)}$ as needed, line 148 runs the subroutine KPLAYS, and lines 149–150 calculate the radial stress increment in the deviatoric plane $∆\rho_{s}$ and the radial stress in the deviatoric plane $s_{\rho }^{\left(2\right)}$. Lines 151–165 update the stress tensor $\sigma_{ij}^{\left(2\right)}$ and, finally, lines 167–171 set the variable EXI to correctly account for Section 2.5 in the calculation of the consistent material Jacobian.

Lines 166 and 167 are executed optimally, as written in Figure 8. However, we found that the analysis under the displacement control in combination with variable temperatures and numerous and small finite elements could not always be completed successfully. We usually achieved convergence by commenting out line 166 and replacing EG2MAX with ALPHA2 in line 167. The side effect is a bit longer calculation time due to more iterations. A deeper understanding of the subject is still necessary.

**Figure 9:**
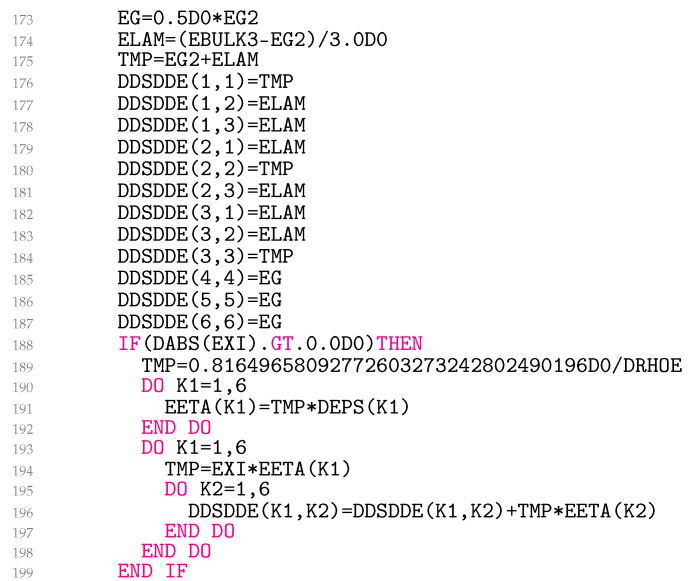
Coding of consistent material Jacobian.

**Figure 10:**
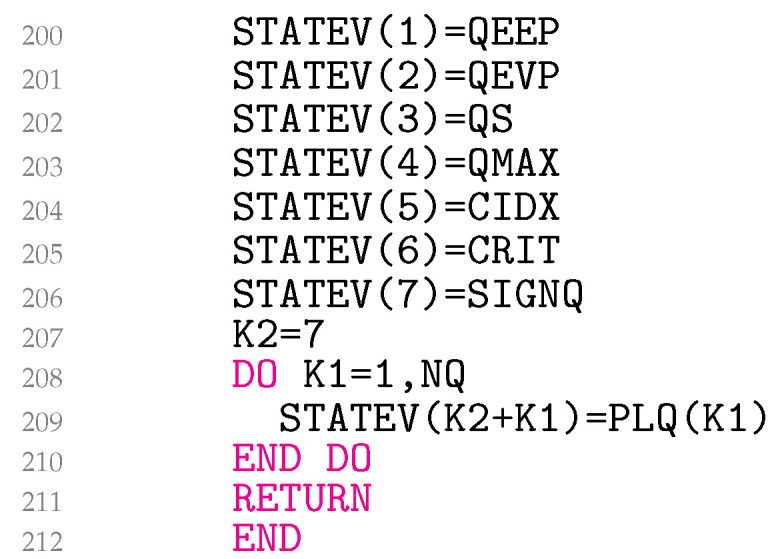
Writing solution-dependent state variables.

In lines 173–187 (Figure 9), Equation (32) is coded, and in lines 188–199, the third summand of Equation (29) is added to the material Jacobian for non-zero EXI. The variable EETA is $\eta_{ij}$. Equations (29) and (32) represent simetric matrices. UMAT needs the material Jacobian for engineering shear strains. Unfortunately, it turns out that the engineering shear strains cause the originally symmetric matrix to become asymmetric. It is worth noting that this problem is not related to the model presented, but, rather, is specific to Abaqus. As a result, lines 189–198 follow the same logic as used in the Abaqus help files for coding kinematic hardening plasticity. Finally, in lines 200–210 (Figure 10), the variables QEEP, QEVP, QS, QMAX, CIDX, CRIT, SIGNQ, and PLQ are written to the solution-dependent state variables.

### 3.6. Advantages and Limitations of the Current Implementation

The advantages of the multiaxial Prandtl operator approach include a high computational power of the algorithm and convenient options for online calculation of quantities that are typically gained by post-processing of the simulated stress-strain results. The computational power originates from the closed-form solution of the temperature-dependent elasto-viscoplastic material response and structured access of both the material properties and solution-dependent variables during the simulation. Convenient post-processing of the simulation results originates from tensor-to-scalar conversion, i.e., the approach internally converts the multiaxial stress–strain states into uniaxial stress–strain states, which makes fatigue damage, creep damage, or dissipated energy easily and quickly calculable during the simulation. However, the limitations of the approach currently include a plateau on the stress–strain curve that may lead to numerical instabilities. Under isothermal conditions, no convergence problems have occurred so far. Under variable temperature conditions, the analysis sometimes may not converge, as the sign of the radial elastoplastic strain increment $∆\rho_{e}$ may start fluctuating. It can currently be controlled as explained for the subroutine KSIGN and replacing variable EG2MAX with ALPHA2 within the subroutine UMAT. Nevertheless, the solution of this limitation is a part of active research.

## 4. Examples

**Figure 11:**
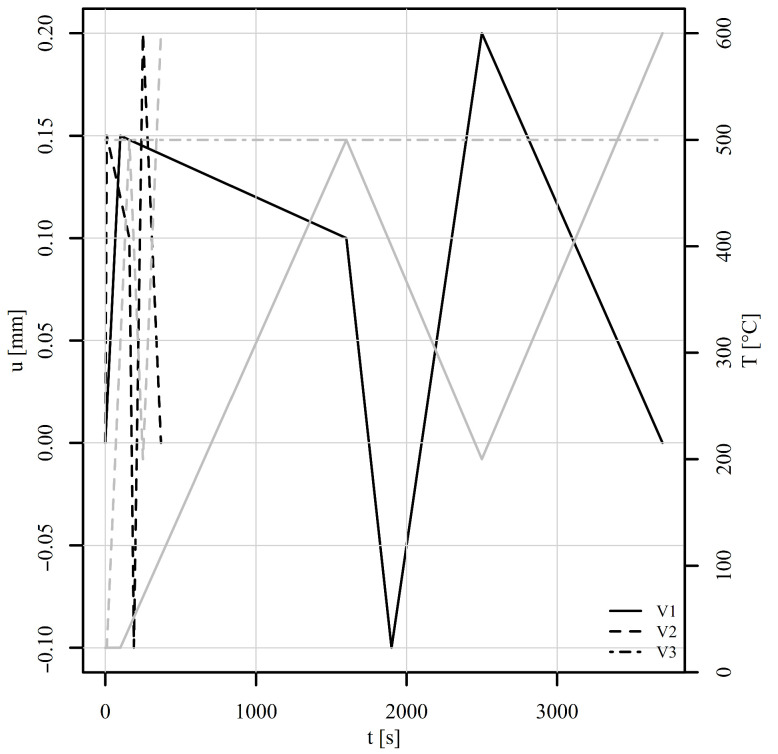
Thermomechanical histories of the tension–compression loads in a displacement-controlled perforated plate. The black lines represent the mechanical load and the grey lines the thermal load.

**Figure 12:**
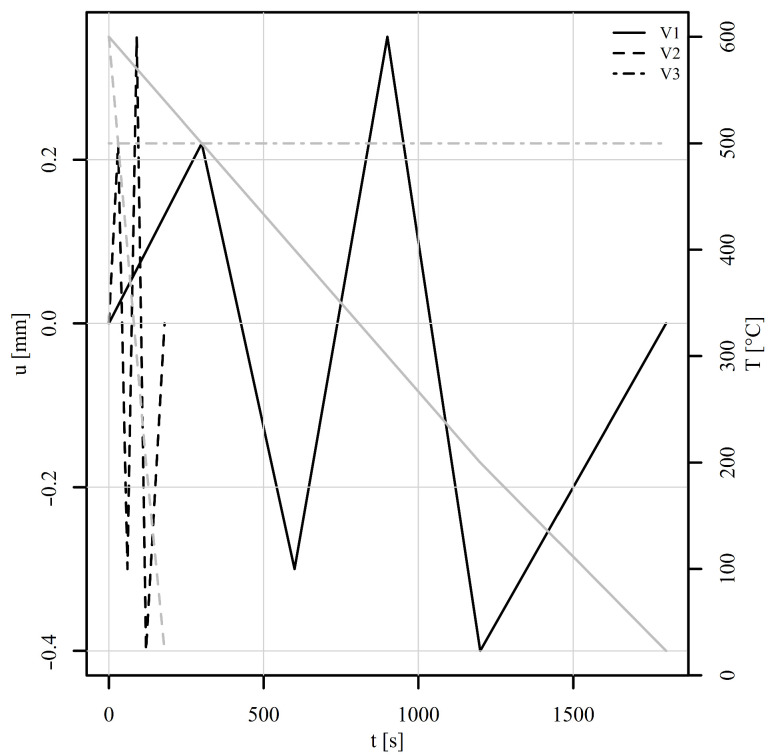
Thermomechanical histories of the shear loads in a displacement-controlled perforated plate. The black lines represent the mechanical load and the grey lines the thermal load.

A perforated plate consisting of 15,063 finite elements of linear type C3D8 and 20,872 nodes was subjected to six variable thermomechanical load histories, shown in Figure 11 and Figure 12. Tension–compression and shear loads were considered. For each load, three variants V1, V2, and V3 were investigated. Variant V1 represents a variable mechanical and thermal load (solid lines). Variant V2 differs from V1 only in the loading rate, which is 10 times higher than for V1 (dashed lines). Variant V3 shares the same mechanical load as V1 but is conducted under constant temperature conditions (represented by the dot-dashed line). All simulations were carried out under displacement control. The boundary conditions considered the displacement of the top edge of the perforated plate whilst the bottom edge remained fixed throughout the analyses. The time stepping schemes for variants V1 and V3 were set so that the temperature change for variant V1 could be also achieved experimentally using a coil or a high-power furnace. The time stepping scheme for V2 was set purely to observe the differences in simulated stress–strain fields between variants V1 and V2 due to the loading rate. The mesh convergence analyses were not performed, as the purpose of the examples was to show the working principle of the implemented method.

To ensure an accurate reproduction of the results of this section, Supplementary Material consisting of plate_tension_compression.inp, plate_shear.inp for variants V1, V2, and V3 are provided.

In addition, the R [61] files material.R and functions.R are provided to facilitate the reproduction of the *USER MATERIAL section of the *.inp files from Table 1. Symbols *T*, $R_{m}$, *E*, $K^{\prime }$, $n^{\prime }$, *k*, *K*, and *N* stand for the temperature, the ultimate strength, the Young’s modulus, the Ramberg–Osgood’s cyclic hardening coefficient, the cyclic hardening exponent, the cyclic yield stress, the Norton’s coefficient, and the Norton’s exponent, respectively. We placed both *.R files in the working directory. The R code allows the calculation of material constants for use in a user-defined mechanical model in Abaqus from known Ramberg–Osgood parameters [62] and the parameters of the law of perfect viscoplasticity with elastic domain. The material constants are stored in the file material.txt and should be copied manually into the *.inp files. The input files can then be executed directly from the Abaqus Command, e.g., with abaqus job=plate_shear_V1 inp=plate_shear_V1.inp user=umat_Prandtl1D_creep.f cpus=6, where umat_Prandtl1D_creep.f contains the code from Section 3.

It must be emphasised here that the material constants for the *.inp files can be constructed for many other elastoplasticity and viscoplasticity laws. However, this may require significant changes in the *.R files but only minor changes in the UMAT subroutine. Of course, there are other ways to construct *USER MATERIAL for the UMAT subroutine.

### 4.1. Perforated Plate Under Tension–Compression Loading

**Figure 13:**
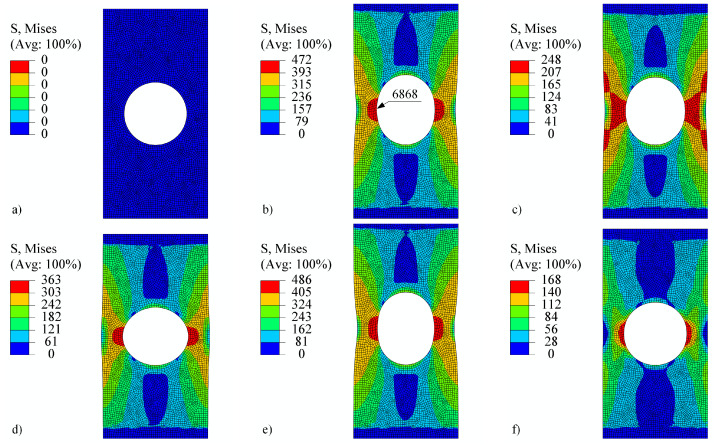
Perforated plate under tension–compression loading (**a**) at the beginning, (**b**) at the end of step 1, (**c**) at the end of step 2, (**d**) at the end of step 3, (**e**) at the end of step 4, and (**f**) at the end of step 5. The stress is given in MPa. The shape change is magnified by a factor of 25.

**Figure 14:**
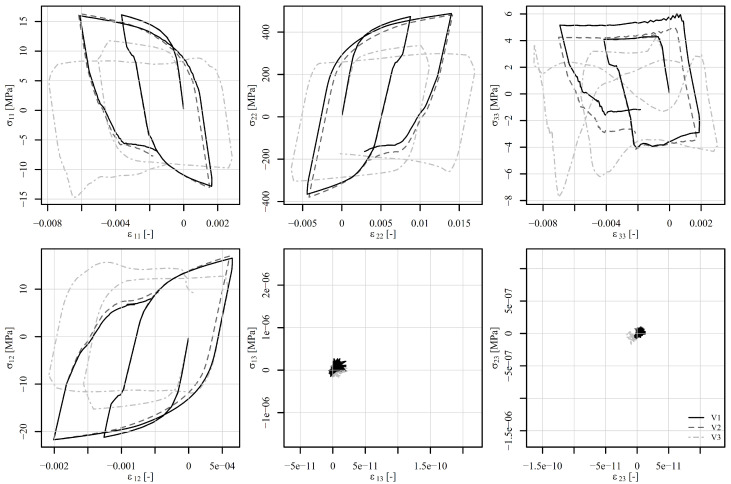
Components of the stress–strain tensor in element 6868 under tension–compression loading of a displacement-controlled perforated plate.

The perforated plate was first subjected to tension–compression loading as shown in Figure 11 (also in [50]). The loading consists of five steps. In the first step, the displacement of the top edge of the perforated plate increases rapidly to 0.15 mm, while the temperature is maintained at 23°C for V1 and V2 and at 500°C for V3. The von Mises stresses on a deformed shape at the end of step 1 are shown in Figure 13b. Although high stress values appear due to low temperature, the highest stresses are concentrated around the perforation. The maximum stress is observed at element number 6868. Figure 14 shows the stress–strain response at this element. It is immediately noticeable that $\varepsilon_{13}$, $\sigma_{13}$ and $\varepsilon_{23}$, $\sigma_{23}$ are zero for all steps. They therefore require no further explanation. The tensor elements 11, 12, and 33 are about an order of magnitude lower than elements 22. It seems feasible to explain the results only for $\varepsilon_{22}$, $\sigma_{22}$. Observing V1 and V2 in the first step reveals that different strain rates have no impact on the stress–strain response. At room temperature, time-dependent phenomena remain inactive. However, V3 already differs from V1 and V2 in this first step due to its pronounced viscoplasticity.

In step 2, the displacement drops to 0.1 mm and the temperature rises to 500°C. The von Mises stresses at the end of the step are best seen in Figure 13c. It can be noticed that the stress values are lower than at the end of step 1, but the area of high stresses around the perforation is considerably larger. The difference in strain rate between V1 and V2 is not yet significant. In step 3, the displacement drops to −0.1 mm and the temperature to 400°C. Here, the stress–strain response for V1 and V2 begin to diverge. The von Mises stresses at the end of the third step are shown in Figure 13d. They are again concentrated around the perforation at this point in compression. V3 is completely different from V1 or V2 because the temperature is high and viscoplasticity is pronounced in all steps. In step 4, the displacement increases to 0.2 mm and the temperature drops further to 200°C. Here, too, we see the expected differences between V1 and V2. In other words, a higher strain rate leads to a lower viscoplasticity. In the final fifth step, the displacement drops to 0 mm and the temperature rises to 600°C. The stress–strain response in Figure 14 shows a stiff response due to the drop of temperature and high mechanical load at the end of step 4. Residual compressive stresses $\sigma_{22}$ reach almost −200 MPa at the end of step 5, although the displacement of the perforated plate equals 0 mm. Figure 13e shows the von Mises stresses at the end of step 4, and Figure 13f shows the residual stresses at the end of step 5.

### 4.2. Perforated Plate Under Shear Loading

**Figure 15:**
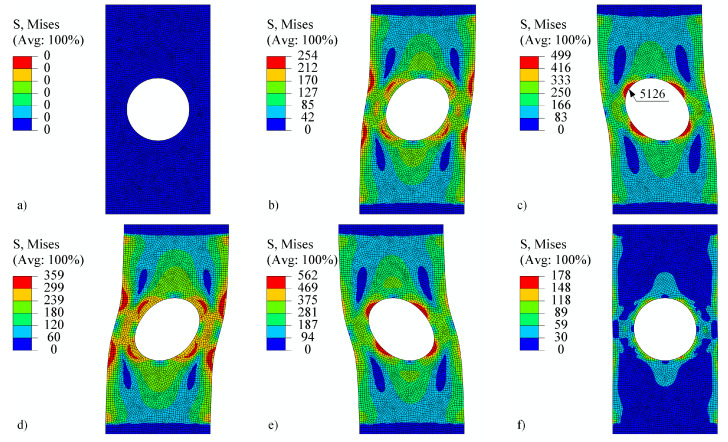
Perforated plate under shear loading (**a**) at the beginning, (**b**) at the end of step 1, (**c**) at the end of step 2, (**d**) at the end of step 3, (**e**) at the end of step 4, and (**f**) at the end of step 5. The stress is given in MPa. The shape change is magnified by a factor of 25.

**Figure 16:**
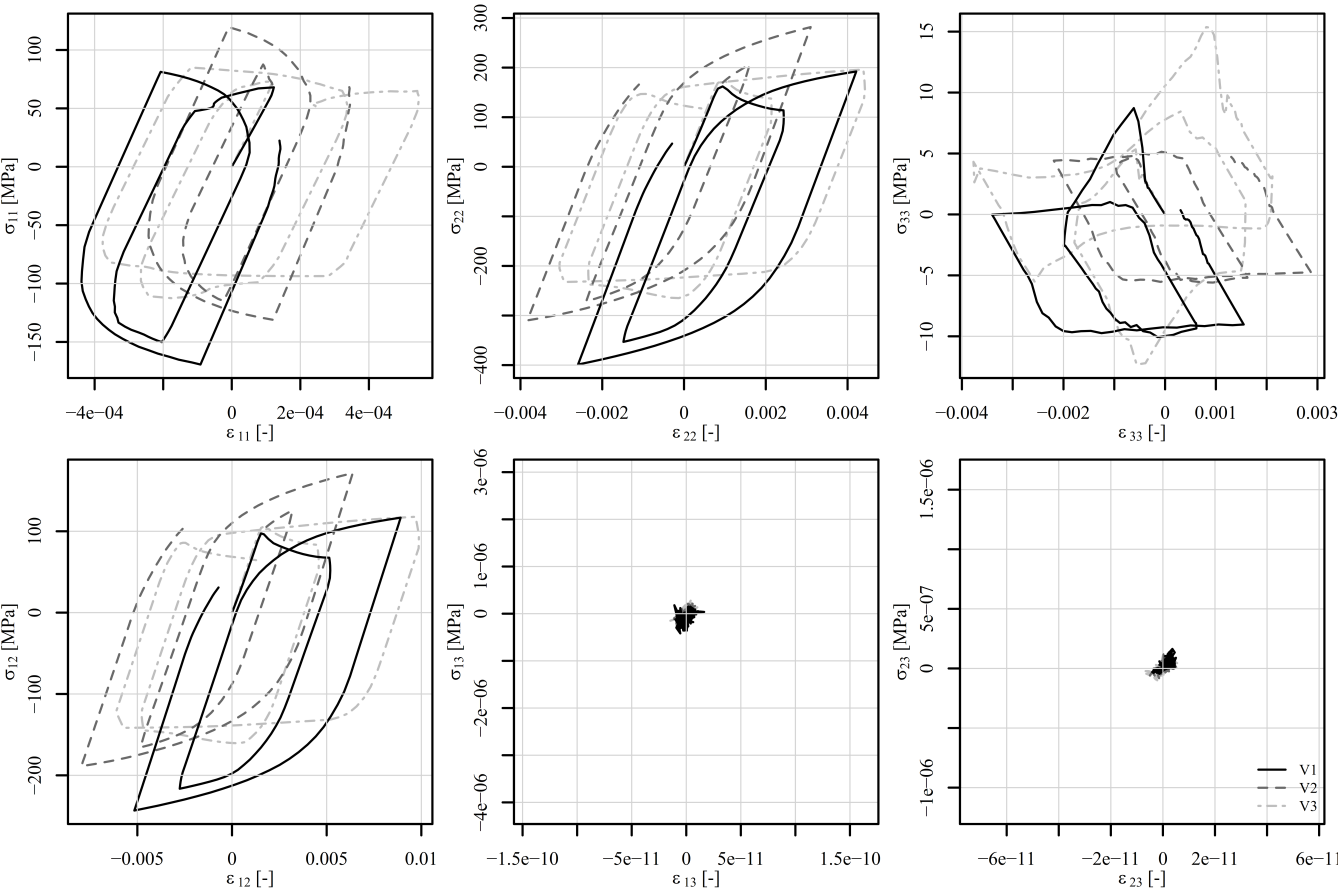
Components of the stress–strain tensor in element 5126 under shear loading of a displacement-controlled perforated plate.

Next, the perforated plate was subjected to a five-step alternating shear loading, as shown in Figure 12. The von Mises stresses of the deformed shapes are shown in Figure 15. Figure 15a shows the initial stress state, while Figure 15b to Figure 15f show the stress states at the end of steps 1 to 5. It can be observed that the position of the most heavily stressed element shifts over time. It is either concentrated around the perforation or additional peaks appear on the vertical edges around the perforation. The stress–strain response is observed at element 5126, where the von Mises stress reaches its maximum. The corresponding components of the stress–strain tensor are shown in Figure 16. The tensor elements 13, 23, and 33 can be omitted from the explanation. It seems sufficient to explain tensor elements 22 further, since elements 11 and 12 show similar behaviour.

At the beginning of the analysis, the temperature is 600°C for load signals V1 and V2. In step 1, the displacement reaches 0.22 mm and the temperature drops to 500°C. From the observation of $\varepsilon_{22}$, $\sigma_{22}$ in Figure 16, it can be seen that the strain rate plays a significant role. While V1 shows a large effect of viscoplasticity, V2 remains almost elastoplastic. In step 2, the displacement changes to −0.3 mm and the temperature drops to 400°C. In this step, the effect of viscoplasticity is still present. In the next steps, however, the temperature linearly approaches the room temperature of 23°C. The effect of viscoplasticity thus decreases steadily. In step 3, the displacement reaches 0.35 mm, in step 4, it changes to −0.4 mm, and in step 5, it attains 0 mm. The stress–strain responses of V1 and V2 are thus similar, although shifted due to viscoplastic effects in steps 1 and 2. The stress–strain response of V3 remains highly viscous due to constant temperature of 500°C throughout the load history. The residual stresses at the end of the analysis are depicted in Figure 15f.

### 4.3. Model Validation

The results of the simulations using the multiaxial Prandtl operator approach for the examples (Section 4) are finally compared to the results of the simulations under equal loading conditions using a reference two-layer viscoplasticity model from the standard Abaqus library of constitutive models. The material parameters of the reference model have been considered as reported in [50] for the 10 CrMo 9 10 material.

**Figure 17:**
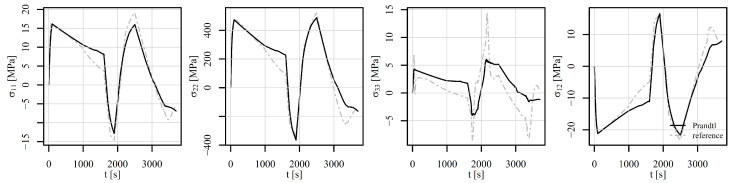
Comparison of the stress tensor in element 6868 under tension–compression loading V1 of a displacement-controlled perforated plate.

**Figure 18:**
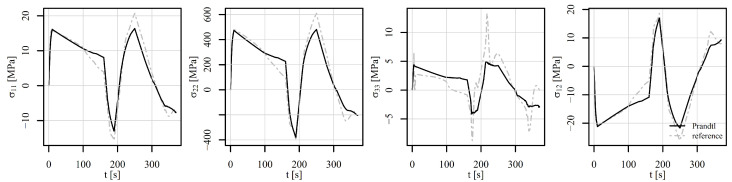
Comparison of the stress tensor in element 6868 under tension–compression loading V2 of a displacement-controlled perforated plate.

**Figure 19:**
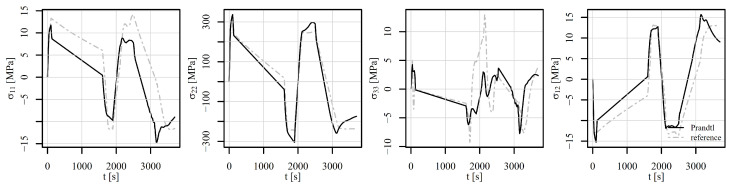
Comparison of the stress tensor in element 6868 under tension–compression loading V3 of a displacement-controlled perforated plate.

Figure 17, Figure 18 and Figure 19 show the comparison of the stress tensor in element 6868 for the tensile–compressive load histories V1, V2, and V3, respectively. Similar trends of the simulation results can be observed with some differences mostly due to temperature-dependent behaviour of the models. Consequently, the highest discrepancies for stress components $\sigma_{11}$ and $\sigma_{22}$ in Figure 17 and Figure 18 can be noticed after 1000 s and 100 s, respectively, where the highest temperature gradient during the loading is applied. The differences are therefore smaller for stress components $\sigma_{22}$ in Figure 19, where constant temperature loading is considered.

**Figure 20:**
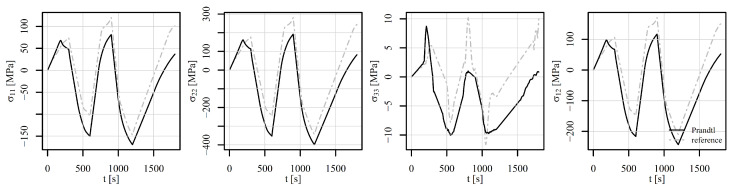
Comparison of the stress tensor in element 5126 under shear loading V1 of a displacement-controlled perforated plate.

**Figure 21:**
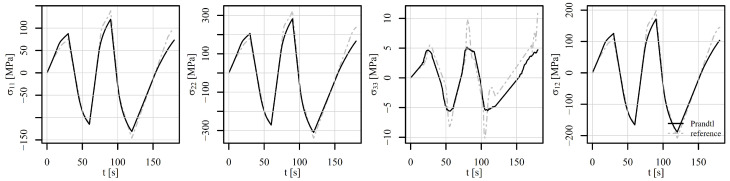
Comparison of the stress tensor in element 5126 under shear loading V2 of a displacement-controlled perforated plate.

**Figure 22:**
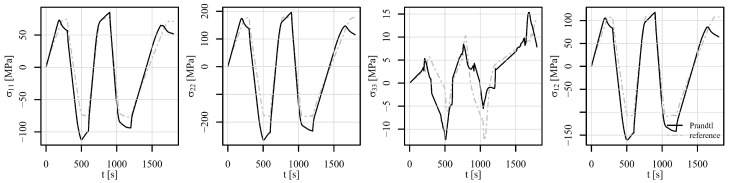
Comparison of the stress tensor in element 5126 under shear loading V3 of a displacement-controlled perforated plate.

Figure 20, Figure 21 and Figure 22 show the comparison of the stress tensor in element 5126 for the shear load histories V1, V2, and V3, respectively. A slight shift of the signals can be observed for the stress components $\sigma_{11}$, $\sigma_{22}$, and $\sigma_{12}$ for all the load histories, but the trends of the stress signals are considerably similar between the compared models. The highest discrepancy amongst the simulated results is obtained for the stress component $\sigma_{33}$, but the absolute values of this component have to be considered, which do not exceed 10 MPa.

## 5. Conclusions

The motivation behind this paper is to provide interested readers with the code that allows a convenient implementation of the recently developed temperature-dependent elasto-viscoplastic multiaxial Prandtl operator approach within the Abaqus finite element solver. The process of translating the equations of this approach into a functional and stable code proved to be lengthy and tedious.

Extensive code testing have shown that the presented approach is numerically stable as long as no physical material limits are reached, such as the plateau of the stress–strain curve. It must also be emphasised that within a single step, if the temperature changes, the displacement or force must also change, at least by a few percent. Otherwise, the analysis may not converge. The reasons for such behaviour are not yet clear. Under isothermal conditions, no convergence problems have occurred so far.

The most important open issue, which has also not yet been fully understood, is the sign of the radial elastoplastic strain increment $∆\rho_{e}$. It depends on the third deviatoric elastoplastic strain-invariant increment, the calculation of which is trivial. However, it has been shown that the calculation can lead to different signs even if two integration points of the same element are considered. When integration points in different elements are observed, the problem becomes even more expressed. It was observed that when Equation (13) is used directly to determine the sign of $∆\rho_{e}$, convergence problems are very likely. The reasons for this have not yet been clarified, even though a lot of effort has been invested in solving the problem. On the contrary, the subroutine KSIGN has proven to be quite good at determining the sign. In its current form, however, it is still not optimal. It seems that it would be better to define the sign outside of UMAT, but Abaqus does not yet offer a way to do this.

Nevertheless, the multiaxial Prandtl operator approach represents the closed-form solution for temperature-dependent elasto-viscoplastic simulations. In [49], it is shown that, compared to the Besseling model [48] provided in Abaqus, a savings of up to 40% in computational time can be expected. The second, even more important advantage of the multiaxial Prandtl operator approach is the conversion of tensor quantities into scalars. This means that the approach internally converts the multiaxial stress–strain states into uniaxial stress–strain states. The signed effective elastoplastic strain $\varepsilon_{\mathrm{ep},\mathrm{eff}}^{\left(2\right)}$, the signed effective viscoplastic strain $\varepsilon_{\mathrm{vp},ij}^{\left(2\right)}$, and the signed effective stress $\sigma_{\mathrm{eff}}^{\left(2\right)}$ are available. This means that fatigue damage, creep damage, dissipated energy, etc., can be calculated continuously at any time with the available uniaxial methods. This is generally not the case with other methods. Only mechanical strains are considered in the paper, but adding thermal strains is trivial and is explained in the Abaqus documentation. Finally, the authors believe that the multiaxial Prandtl operator approach can also be extended to isotropic hardening [63] and anisotropy, which remains an open goal for the future.

## Figures and Tables

**Table 1:** Material parameters of 10 CrMo 9 10, heat treatment 930°C/1.5h air, 710°C/1.5h air, 680°C furnace [50].

*T* [°C]	$R_{m}$ [MPa]	*E* [MPa]	$K^{\prime }$ [MPa]	$n^{\prime }$ [-]	*k* [MPa]	Nln(K) [MPa]	*N* [-]
23	627	210,000	842.0	0.1180	862.8	40.08	4.8380
300	553	204,100	773.0	0.1190	374.2	17.56	1.3324
400	530	187,800	688.0	0.1029	296.6	14.13	0.8018
500	466	184,800	500.3	0.0773	238.9	11.03	0.3034
600	344	162,000	331.9	0.0583	194.8	9.47	0.0788

## Data Availability

The original contributions presented in this study are included in the article/supplementary material. Further inquiries can be directed to the corresponding author(s)

## Supplementary Materials

The following supporting information can be downloaded at: https://www.mdpi.com/article/10.3390/ma18112512/s1: The complete computer code of the UMAT subroutine for Abaqus, the input files of the examples, and the files to prepare the material parameters.
